# Epileptogenesis After Stroke: Current Insights Into Molecular and Structural Mechanisms

**DOI:** 10.7759/cureus.101385

**Published:** 2026-01-12

**Authors:** Meet Popatbhai Kachhadia, Sarah Codreanu, Imad Sibhai, Usmaan Topiwala, Pathan Mohmad Rafe Iqbal, Rushi Vaghela, Sunil Chauhan, Juber D Shaikh

**Affiliations:** 1 Neurology, Florida Atlantic University Charles E. Schmidt College of Medicine, Boca Raton, USA; 2 Internal Medicine, Florida Atlantic University Charles E. Schmidt College of Medicine, Boca Raton, USA; 3 Medical Education, Smt. Nathiba Hargovandas Lakhmichand (NHL) Municipal Medical College, Ahmedabad, IND; 4 General Medicine, Smt. Nathiba Hargovandas Lakhmichand (NHL) Municipal Medical College, Ahmedabad, IND; 5 Internal Medicine, New Ahmadi Hospital, Ahmadi, KWT; 6 Internal Medicine, Smt. Nathiba Hargovandas Lakhmichand (NHL) Municipal Medical College, Ahmedabad, IND; 7 Neurology, Prisma Health Residency, University of South Carolina, Columbia, USA

**Keywords:** biomarkers, blood–brain barrier dysfunction, epileptogenesis, neuroinflammation, post-stroke epilepsy

## Abstract

Stroke is among the most common causes of acquired epilepsy in adults, and post-stroke epilepsy (PSE) is a substantial driver of long-term disability. Epileptogenesis after stroke is not a single event but a prolonged, multi-phase process in which structural injury (neuronal loss, gliosis, blood-brain barrier (BBB) dysfunction, and maladaptive synaptic remodeling) interacts with molecular programs, including excitotoxicity, inflammation, oxidative stress, and epigenetic reprogramming, to create a persistently hyperexcitable network. Recent advances in neuroimaging, electrophysiology, and molecular profiling have yielded a growing set of candidate biomarkers (lesion topology and volume, metabolic and microstructural imaging signatures, early electroencephalographic abnormalities, and blood or CSF-derived proteins and microRNAs). Although none has yet been validated for routine clinical use, a multimodal, longitudinal biomarker strategy could enable risk stratification and serve as a surrogate endpoint for anti-epileptogenic trials. Therapeutic development remains focused largely on seizure suppression, but anti-inflammatory, antioxidant, metabolic-epigenetic, mitochondrial, and neuromodulatory approaches demonstrate promise in preclinical and translational studies. A clearer understanding of how structural damage and molecular signaling interlock over time, paired with pragmatic biomarker frameworks, offers a path toward prevention that may reduce the burden of epilepsy after stroke.

## Introduction and background

Post-stroke seizures and post-stroke epilepsy (PSE) are frequent and clinically consequential. In population-based and health system studies, stroke is a leading cause of adult-onset epilepsy, and the burden is particularly high in older adults; late seizures after both ischemic stroke and intracerebral hemorrhage (ICH) contribute meaningfully to morbidity and healthcare utilization [1,2]. The risk is amplified by cortical involvement, larger lesion volumes, and hemorrhagic etiologies, features that align with guideline-recognized predictors of early and late seizures in acute cerebrovascular disease [3,4]. Conceptually, “epileptogenesis” denotes the process by which a previously non-epileptic brain acquires a stable propensity for spontaneous seizures. In stroke, that process begins with the index ischemic or hemorrhagic injury and continues through a latent period of network remodeling into a chronic, self-reinforcing ictogenic state [5,6].

The field has moved beyond a simplistic latent-period model to a more dynamic, longitudinal view. Subclinical and electrographic seizures can precede the first clinical event, periodic and rhythmic EEG patterns may evolve over days to weeks, and the frequency and severity of events may escalate over time, observations that motivate continuous and early EEG strategies in selected patients and inform how we conceptualize mechanistic windows for intervention [7-9]. These evolving insights, in turn, underscore why biomarkers must be sensitive to time and trajectory rather than single snapshots. Blood and CSF analytes, quantitative EEG (qEEG) metrics, and structural/functional MRI markers exemplify complementary “views” of epileptogenesis that could be combined for prediction and for trial endpoints [10]. Because stroke is heterogeneous, with differing vascular mechanisms, lesion locations, and systemic comorbidities, stroke-specific frameworks are essential. Vascular risk, sleep, and small-vessel disease biology, as well as neuroinflammation, all shape post-stroke network vulnerability [11]. Despite advances in acute stroke care and secondary prevention, there are currently no validated strategies to prevent post-stroke epileptogenesis, and clinical decision-making remains largely reactive rather than preventive. As survival after stroke improves and the population ages, the burden of post-stroke seizures and PSE continues to grow, underscoring the need for timely identification of patients at highest risk. This review is therefore timely in synthesizing recent advances in molecular and structural mechanisms, emerging biomarkers, and evolving network-level concepts to provide an integrated framework that can inform risk stratification, guide future interventional trials, and identify opportunities for disease-modifying strategies.

Methods: scope and approach

This narrative review synthesizes peer-reviewed evidence published between January 2021 and September 2025, focusing on epileptogenesis after stroke, with emphasis on structural and molecular mechanisms, candidate biomarkers, and therapeutic implications. We searched PubMed/MEDLINE, Embase, Web of Science, and the Cochrane Library using combinations of terms related to stroke (ischemic stroke, intracerebral hemorrhage), post-stroke seizures/PSE, epileptogenesis, biomarkers (EEG, imaging, blood, CSF), inflammation/oxidative stress, epigenetics, and neuromodulation; reference lists of key reviews and guidelines were hand-searched to identify additional studies.

Eligible evidence included original studies in adult humans (≥18 years) and translational animal models directly informing mechanisms, biomarkers, or preventive strategies; high-quality narrative or systematic reviews were used to contextualize and triangulate findings, where appropriate. We prioritized ischemic stroke and intracerebral hemorrhage; aneurysmal subarachnoid hemorrhage was considered when mechanistic or biomarker insights were directly pertinent to post-stroke epileptogenesis. We excluded pediatric populations, non-stroke etiologies of acquired epilepsy, case reports, conference abstracts without adequate methodology, and non-English publications. Titles/abstracts were screened, followed by full-text appraisal, when overlapping reports from the same cohort were identified; the most recent or methodologically comprehensive publication was retained to avoid double-counting.

We excluded pediatric stroke cohorts. This decision was deliberate, as pediatric stroke differs from adult stroke in epidemiology, etiologic spectrum (e.g., arteriopathies, congenital heart disease, perinatal factors), brain maturation state, and seizure mechanisms, and PSE appears to be more frequent in children than adults. As a result, the incidence estimates, biomarker profiles, and risk models synthesized here should not be extrapolated to pediatric stroke survivors. Dedicated reviews and prediction tools are needed for the pediatric population, and our findings should be interpreted as applying primarily to adult-onset stroke.

Given the heterogeneity in populations, endpoints (early vs. late seizures, epilepsy incidence), EEG acquisition/definitions, imaging protocols, and biomarker assay methods, no formal meta-analysis was performed; instead, study quality and risk of bias were assessed qualitatively with attention to design, sample size, adjustment for confounders, outcome ascertainment (including continuous EEG where relevant), and assay validity. Definitions and terminology were anchored to contemporary AHA/ASA guidance for ICH and ACNS standardized EEG terminology to ensure consistency across studies.

## Review

Pathophysiology of epileptogenesis after stroke

Epileptogenesis unfolds across phases that blend into one another. An acute injury window (minutes to days) features glutamate-driven excitotoxicity, ionic disequilibria, metabolic crisis, and early innate immune activation. A subacute or latent remodeling period (weeks to months) consolidates network hyperexcitability via synaptic and axonal rewiring, channelomic reprogramming, and glial-vascular interactions. Ultimately, a chronic state emerges in which spontaneous recurrent seizures stabilize circuits into a pro-ictogenic attractor and further remodel synapses, dendrites, and glia [5,6]. Cross-insult commonalities shared with trauma and status epilepticus support this phased model, while overlaying controls, epigenetic and transcriptional programs, neurovascular and glymphatic coupling, and cerebrovascular comorbidity, modulate its tempo and trajectory [6,12].

Structural mechanisms

Structural pathology begins at the index insult. In ischemia, necrosis dominates the infarct core, while apoptosis and sublethal injury extend into the penumbra. In hemorrhage, mass effect, heme and iron toxicity, and perihematomal edema create related and overlapping injury patterns. Selective vulnerability of inhibitory interneurons, particularly parvalbumin-positive, fast-spiking GABAergic cells, disrupts local circuit balance and reduces feedforward and feedback inhibition, lowering the seizure threshold in surviving cortex and hippocampus. Astroglial and microglial responses then remodel the extracellular milieu: reactive astrogliosis (with GFAP upregulation) alters potassium buffering and glutamate clearance, while activated microglia release cytokines and reactive oxygen species (ROS) that aggravate synaptic dysfunction and neuronal stress [5].

Blood-brain barrier (BBB) disruption is central in both ischemic and hemorrhagic strokes. Leakage of serum proteins, such as albumin and fibrinogen, into the parenchyma triggers astrocytic transforming growth factor-β signaling, perturbs Kir4.1-mediated K+ buffering, and contributes to loss of aquaporin-4 (AQP4) perivascular polarity. These events not only amplify glial reactivity and excitatory signaling but also impair perivascular (glymphatic) clearance of solutes, coupling BBB injury to persistent neuroinflammation and extracellular accumulation of glutamate and potassium. After a stroke, this BBB-glymphatic axis likely interacts with cerebral small-vessel disease (CSVD) and sleep fragmentation to prolong the “tail” of injury and extend the window in which epileptogenesis can take hold [12]. Over weeks to months, aberrant sprouting, dendritic remodeling, and formation of new excitatory synapses increase network gain and the propensity for hypersynchronous discharges. Maladaptive reorganization in hippocampal and neocortical circuits has been documented in experimental models and post-mortem studies and maps well to the clinical predilection of cortical lesions for generating late seizures [5].

Ischemic stroke-related epileptogenesis

In ischemic stroke, the epileptogenic cascade begins with focal hypoperfusion and energy failure, producing an infarct core characterized by necrosis and a surrounding penumbra with apoptosis and sublethal injury. Excess glutamate release and failure of ionic homeostasis in the ischemic territory drive NMDA/AMPA-mediated excitotoxicity, intracellular calcium overload, and mitochondrial dysfunction. Selective vulnerability of fast-spiking, parvalbumin-positive GABAergic interneurons in peri-infarct cortex and hippocampus results in disproportionate loss of inhibition and a shift toward excitatory dominance. Over days to weeks, BBB disruption, albumin extravasation, and activation of astrocytic TGF-β signaling alter potassium buffering and glutamate clearance, while reactive astrogliosis and microgliosis sustain local inflammation and oxidative stress. These processes promote aberrant axonal and dendritic sprouting in peri-infarct cortex, reorganization of thalamo-cortical and hippocampal circuits, and channelomic remodeling (including KCC2 downregulation and HCN/Na+ channel reprogramming), progressively lowering seizure threshold in structurally altered but surviving networks [5,12].

Hemorrhagic stroke-related epileptogenesis

In ICH and other hemorrhagic cerebrovascular insults, the initial drivers of epileptogenesis differ in emphasis. Sudden extravasation of blood into the brain parenchyma produces acute mass effect, increased intracranial pressure, and mechanical distortion of adjacent cortex. The clot and perihematomal region expose neural tissue to hemoglobin breakdown products, iron-mediated oxidative stress, thrombin, and other pro-inflammatory mediators that directly depolarize neurons and activate microglia and astrocytes. Perihematomal edema and local BBB disruption further amplify ionic disequilibria and permit sustained infiltration of serum proteins that engage astrocytic TGF-β pathways. Over time, resorption of the hematoma leaves behind a hemosiderin-laden, gliotic cavity at the cortical-subcortical interface, which behaves as a chronic irritative focus. Cortical location, large hematoma volume, and intraventricular extension, the core components of CAVE-family scores, capture this convergence of structural distortion, blood-product toxicity, and gliosis that underpins the higher seizure propensity seen after lobar ICH compared with deep or purely ischemic lesions [5,12].

Channelomic and ionic homeostatic shifts

Structural rewiring is accompanied by enduring changes in the expression and function of ion channels and transporters. Downregulation of the neuronal KCC2 chloride exporter and relative upregulation of NKCC1 in the injured cortex can attenuate the hyperpolarizing effect of GABAergic currents, rendering inhibition less effective or even paradoxically depolarizing. Altered HCN-mediated sag currents and remodeling of voltage-gated sodium channel subtypes further bias dendrites toward burst-prone dynamics and accelerate population synchrony. These channelomic shifts both reflect and reinforce inflammatory signaling and metabolic stress, tightening the feedback loop that sustains hyperexcitability [6].

Molecular mechanisms

The molecular arm of epileptogenesis begins with excitotoxicity. Excess glutamate release during ischemia and early reperfusion overactivates NMDA and AMPA receptors, permitting calcium influx that drives protease activation, mitochondrial dysfunction, and free radical production. Oxidative and nitrosative stress injures membranes, impairs energy metabolism, and modifies channel behavior in ways that potentiate hyperexcitability [6]. Neuroinflammation is a parallel and durable driver: interleukin-1β (IL-1β), tumor necrosis factor-α (TNF-α), interleukin-6, and other mediators increase in brain tissue and peripheral blood after stroke, alter synaptic transmission by enhancing glutamatergic signaling and reducing GABAergic efficacy, and are amplified by toll-like receptor and NF-κB pathways [10,11]. Epigenetic and transcriptional programs translate transient injury signals into stable, pro-epileptogenic gene expression states. DNA methylation, histone post-translational modifications, and non-coding RNAs, including multiple microRNAs implicated in synaptic function, channel expression, and inflammatory pathways, have been linked to acquired epilepsies in experimental systems and, increasingly, in human studies [6,10]. A particularly intriguing axis is metabolic-epigenetic coupling via histone lysine lactylation. Post-ischemic glycolysis elevates lactate, which can be incorporated into histone lactylation marks that reprogram inflammatory and synaptic transcripts in astrocytes and neurons; in this way, energy metabolism may directly influence durable gene expression patterns permissive of hyperexcitability [13]. Mitochondrial quality control also appears pivotal: mitochondrial fragmentation and calcium overload impair mitophagy, raising ROS production and degrading synaptic integrity. Translational experiments targeting Rho-associated kinase (ROCK2) suggest that restoring mitophagy can normalize network synchrony and shift synaptic vesicle protein 2A (SV2A) imaging signals toward physiological ranges-nominally “drugging” a pathway with measurable, mechanistically aligned surrogate markers [14].

Integrating structure and signaling

In the nervous system, structure and signaling move in lockstep. When the BBB fails, serum proteins enter the parenchyma and trigger astrocytic TGF β pathways, enhancing inflammatory transcription. In parallel, interneuron loss and activity-dependent synaptic reorganization reshape local circuits; those circuit shifts feed back on cytokine tone, ion channel expression, and mitochondrial load. At the bedside, this coupling appears as electrographic instability, interictal discharges, and lateralized periodic discharges along with perfusion and metabolic changes, such as periictal hypoperfusion on arterial spin labeling MRI, and slow, directional volumetric change. These signatures cohere with recognized high-risk phenotypes, particularly cortically dominant infarcts and intraparenchymal hemorrhage [7,8,12,15]. Because they are concrete and time-stamped, they lend themselves to surveillance protocols and to the design of biomarker-enriched prevention trials.

Biomarkers and predictors of post-stroke epileptogenesis

For definitions used in clinics and studies, early post-stroke seizures occur within seven days of stroke onset, while late seizures occur after seven days. PSE is typically diagnosed following two unprovoked late seizures, or after a single late seizure when the estimated risk of recurrence is high. These working rules align with established guidelines and EEG terminology frameworks and track biological epochs: the first week encompasses acute excitotoxic and inflammatory cascades; the ensuing weeks to months represent a latent remodeling phase; and later time points reflect a more stable chronic phase [3,8]. Electrographic abnormalities can blur boundaries; acute symptomatic seizures may shade into nonconvulsive status epilepticus, so criterion-based EEG interpretation, anchored in clinical context, is essential for accurate classification and timely treatment.

Structural and functional imaging

Structural neuroimaging is the most practical predictor set in routine care. Cortical involvement is strongly linked to late seizures, and larger lesions, especially in the frontal or temporal lobes, carry greater epileptogenic risk [4]. In ICH, hematoma location, volume, and intraventricular extension shape risk; validated tools such as CAVE, CAVS, and LANE (including modified versions) combine these features with early seizures to estimate the probability of late epilepsy and support triage to prolonged monitoring and closer follow-up [16,17]. In ischemic stroke, SeLECT and its updated SeLECT 2.0 integrate clinical and imaging variables to stratify risk and guide surveillance decisions [18]. In subarachnoid hemorrhage, the RISE score captures hemorrhage burden and relevant complications, illustrating how etiology-specific models sharpen prediction [19]. Beyond gross lesion metrics, advanced MRI can capture microstructural and metabolic changes that precede clinical seizures. Diffusion tensor imaging can reveal white matter tract disruption and hippocampal microstructural alterations, while volumetric MRI tracks regional atrophy trajectories over months. Fluorodeoxyglucose-PET has demonstrated entorhinal and hippocampal hypometabolism during latency in acquired epilepsy models, and magnetic resonance spectroscopy (MRS) markers (lower N-acetylaspartate as a proxy for neuronal integrity and higher myo-inositol as a glial activation signal) change dynamically after stroke and may correlate with subsequent seizure susceptibility [5,10]. Functional MRI-EEG coupling and perfusion imaging add a further layer: peri-ictal negative ASL patterns have been described in acute SAH with NCSE and may serve as a bridge between physiology and imaging in biomarker panels [15].

Risk factors for post-stroke seizures and PSE

Multiple clinical, radiographic, and electrographic variables consistently stratify the risk of both early post-stroke seizures (within seven days) and late seizures/PSE. Across large cohort studies and recent meta-analyses, hemorrhagic stroke, greater stroke severity, cortical involvement, and hemorrhagic transformation emerge as the most robust predictors. Hemorrhagic etiologies, lobar intracerebral hemorrhage in particular, confer a higher seizure risk than pure ischemic infarction, reflecting the combined effects of acute blood products, mass effect, and perihematomal edema on cortical irritability. Hemorrhagic transformation of ischemic infarcts similarly increases the risk of both early and late seizures. Large lesion volume and higher NIHSS scores correlate with both early electrographic events and subsequent PSE, underscoring the contribution of global injury burden. Cortical involvement, especially in the frontal and temporal regions, is a reproducible risk factor for late seizures and PSE in both ischemic stroke and ICH and is incorporated into widely used prognostic tools. In ischemic stroke, the SeLECT score and its subsequent refinements combine stroke Severity, cortical Location, Early seizures, involvement of the MCA Territory, and aetiologic factors to estimate late seizure risk. In ICH, the CAVE and modified CAVE scores integrate Cortical location, younger Age, hematoma Volume, and Early seizures to predict late seizures. Early clinical or electrographic seizures are themselves among the strongest predictors of PSE: patients who experience acute symptomatic seizures after stroke have a several-fold higher risk of subsequent epilepsy than those without early events. Emerging evidence also implicates younger age (<65 years in several series), anterior circulation infarcts, and selected genetic variants (e.g., polymorphisms in inflammatory or ion-channel genes) as additional modifiers of risk [15,18,19].

Taken together, these data support a pragmatic, tiered approach in which lesion type (ischemic vs hemorrhagic), cortical involvement, lesion size, stroke severity, and early seizures are used to generate a baseline risk estimate that can then be refined by EEG findings and biomarker trajectories.

Electrophysiology: from patterns to quantitative networks

EEG is a natural biomarker arena because it directly samples the electrical substrate of seizures. Interictal epileptiform discharges and lateralized periodic discharges in the early post-stroke period are associated with increased risk of later epilepsy, and standardized terminology improves prognostic consistency across centers [8,20]. Prospective studies highlight the prognostic value of early electrographic abnormalities, including rhythmic and periodic patterns that may be subclinical yet mechanistically important for network priming [21]. Point-of-care EEG, deployed in the emergency department or early in hospitalization, can detect electrographic seizures and NCSE rapidly, providing actionable information during the latent window when neuroinflammation, BBB dysfunction, and synaptic rewiring are in flux [22,23]. In ischemic stroke, early NCSE has been linked to specific clinical phenotypes, such as impaired consciousness, and may portend worse outcomes, reinforcing the need for judicious monitoring strategies [23]. Longer-term, qEEG connectivity metrics and interictal burden trajectories could offer more sensitive, continuous readouts of network evolution from acute to chronic phases [20-22].

Molecular markers in blood and CSF

Circulating and CSF biomarkers promise a dynamic, mechanistically grounded vantage point on epileptogenesis. Panels anchored in neuronal and glial injury (neuron-specific enolase, ubiquitin carboxyl-terminal hydrolase L1, myelin basic protein, GFAP, S100B), BBB integrity, and inflammation (IL-1β, IL-6, TNF-α, high-sensitivity C-reactive protein) have been examined across stroke cohorts, with higher levels generally tracking more severe brain injury, BBB disruption, and glial activation, processes central to epileptogenesis [10]. Cytokine signatures can be complemented by microRNAs that regulate synaptic, inflammatory, and channel-expression programs; several miRNAs implicated in acquired epilepsy overlap with post-stroke miRNA profiles, raising the possibility of shared, portable fingerprints [10]. Evidence from SAH and ischemic cohorts supports a role for CSF inflammatory mediators in electrographic instability, with higher inflammatory burden associated with nonconvulsive seizures [24]. Exploratory work in stress-neuropeptide signaling suggests that circulating neuropeptide-Y correlates with PSE risk, potentially indexing broader neuroimmune-autonomic adaptations after ischemia [25].

GFAP and S100B may peak in the early subacute period, miRNA signatures can persist for weeks, and inflammatory mediators show distinct acute and subacute profiles. The preanalytical variability of assays, differences in sampling intervals, and the need for multi-marker panels that reflect complementary biology are recurring lessons from systematic reviews [10]. Looking ahead, biomarker strategy should be explicitly longitudinal, matching the phased nature of epileptogenesis and the likely windows in which particular biological processes dominate.

Composite, translational, and predictive frameworks

No single measure will fully capture the complexity of post-stroke epileptogenesis. A pragmatic approach blends structural imaging (cortical involvement and lesion burden), early EEG (electrographic seizures, periodic patterns, and qEEG features), and blood/CSF signals of glial activation, BBB injury, and inflammation, all measured serially to create a trajectory-aware risk portrait. In practice, patients with ischemic stroke can be triaged by SeLECT 2.0, with monitoring intensity and ambulatory EEG timing adjusted upward for those with early interictal abnormalities or electrographic seizures and for those with large cortical infarcts. In ICH, modified CAVE-family scores can be augmented by the persistence of interictal activity and by inflammatory biomarkers to trigger closer follow-up [16-18]. In SAH, RISE-stratified monitoring plus perfusion-coupled EEG in deteriorating patients may capture otherwise occult instability [19].

Machine-learning models that integrate EHR features, imaging metrics, and EEG time series are emerging for both ischemic stroke and ICH, with horizons at one and five years; early reports suggest improved discrimination when models are trained on biomarker-augmented features rather than clinical variables alone [26-28]. Genetic and epigenetic information can further refine pretest probabilities. Systematic reviews highlight polymorphisms in inflammatory and synaptic genes associated with seizure risk after acute brain injury, and large biobank analyses indicate that polygenic risk of epilepsy interacts with clinical context to influence PSE susceptibility [29,30]. These layers (structured clinical scores, dynamic physiology, omics, and ML) are complementary rather than competing.

Therapeutic implications and future directions for prevention

Current practice remains predominantly reactive. Outside of specific indications (e.g., treatment of acute symptomatic seizures or NCSE), routine prophylactic antiseizure medication (ASM) after ischemic stroke or ICH is not guideline-supported, and randomized data have not shown durable benefits of blanket prophylaxis on long-term epilepsy risk or functional outcomes [3,31,32]. This gap between mechanistic understanding and preventive options is the central motivation for biomarker-enriched intervention trials.

Anti-inflammatory, antioxidant, and neuroprotective strategies

Anti-inflammatory agents that attenuate IL-1 signaling and related innate immune pathways reduce seizure susceptibility in experimental systems and map naturally onto the inflammatory arm of post-stroke epileptogenesis [6,10,11]. Cyclooxygenase inhibition and other immunomodulators have likewise shown promise in preclinical models, although safety and target engagement must be demonstrated in stroke populations. Antioxidant approaches, including N-acetylcysteine and agents that bolster mitochondrial redox defenses, are biologically plausible, given the prominence of oxidative stress in both ischemia and epileptogenesis; so too are neuroprotective compounds used in acute stroke care, such as minocycline and statins, whose pleiotropic effects include microglial modulation and endothelial stabilization [11].

Interventions that temper excitotoxic cascades, targeting NMDA or AMPA receptor signaling, have yielded mixed results historically, in part due to narrow therapeutic windows and adverse effects. Their future utility may depend on coupling to biomarkers that verify on-target engagement and on dosing schemas that respect phased biology (e.g., excitotoxic dominance early, inflammatory and epigenetic consolidation later).

Metabolic-epigenetic, mitochondrial, and systems-level levers

If a central challenge of prevention is converting transient post-injury signals into a durable deviation away from the ictogenic attractor, then epigenetic control points are attractive. Histone lactylation, as a direct readout of glycolytic flux, provides a mechanistic bridge between energy metabolism and persistent transcriptional states; targeting the writers, readers, or erasers of lactylation, or modulating lactate shuttling itself, could reshape astrocytic and neuronal programs toward anti-epileptogenic profiles [13]. Mitochondrial quality-control pathways offer a second therapeutic foothold. ROCK2 inhibition to restore mitophagy has produced encouraging translational signals, including SV2A-based imaging correlates that could anchor surrogate endpoints alongside electrophysiological readouts [14].

A third lever is systems-level: the BBB-glymphatic-CSVD axis and sleep. Because BBB leak and glymphatic dysfunction appear to prolong inflammatory and metabolic “tails” after stroke, structured sleep assessment (including screening and treatment for obstructive sleep apnea), blood-pressure and lipid optimization, and graded aerobic rehabilitation should be considered disease-modifying adjuncts that may reduce the reservoir of pro-epileptogenic signaling [11,12]. Such pragmatic, high-yield interventions can be layered on top of EEG-guided surveillance in high-risk phenotypes to capture the latent-to-chronic transition earlier.

Neuromodulation and non-pharmacologic approaches

Network-level interventions, such as vagus nerve stimulation, transcranial magnetic stimulation, deep brain stimulation, and emerging noninvasive cortical stimulation paradigms, are established tools for drug-resistant epilepsy and may, if applied during the latent period, aid in “retuning” post-stroke networks away from epileptogenic configurations. Early PSE series and related acquired-epilepsy models suggest feasibility and warrant trials that incorporate qEEG connectivity and cognitive co-endpoints [11]. Remote ischemic conditioning (repeated, transient limb ischemia) has demonstrated neuroprotective effects in other neurological contexts and presents an intriguing, low-cost candidate for modulating inflammation, perfusion, and mitochondrial pathways relevant to epileptogenesis, though it remains to be tested directly for PSE prevention [33].

From biomarkers to trials

A major impediment to preventive therapy development is the absence of validated surrogate endpoints. Waiting for clinical seizures can necessitate large cohorts and long follow-up, blunting the feasibility of early-phase testing. The solution is two-fold. First, adopt rigorously phenotyped, longitudinal biomarker batteries that track glial and BBB injury (e.g., GFAP, S100B), inflammation (IL-1β, IL-6, TNF-α), and electrographic burden (interictal discharges, seizure minutes per day, qEEG connectivity) from acute through subacute phases. Second, integrate these with imaging surrogates such as SV2A PET or volumetric trajectories, where appropriate. With this scaffolding, anti-inflammatory or mitochondrial interventions, for example, could be judged on their ability to normalize specific biomarker trajectories over weeks to months, well before enough clinical seizures accrue to power hard-endpoint analyses [10,14,20-22].

A practical, time-stamped surveillance strategy

In clinical pathways, time and topology should drive surveillance intensity. Patients with large cortical ischemic strokes or lobar ICH qualify as high-risk by lesion characteristics alone; SeLECT 2.0 (ischemia) and modified CAVE-family scores (ICH) can formalize this baseline risk [16-18]. Early EEG (continuous for the first 24-48 hours in critically ill patients or point-of-care EEG in emergency and step-down settings) can identify electrographic seizures or periodic patterns that further escalate risk and justify prolonged monitoring and follow-up [7,8,22,23]. In SAH, RISE-guided triage combined with perfusion-informed MRI in deteriorating patients can unmask NCSE that would otherwise be missed [15,19]. Where feasible, incorporate one or two blood-based markers of glial activation and inflammation during hospitalization and at a subacute clinic visit; abnormal trajectories should prompt ambulatory EEG and closer neurology follow-up. This “score-plus-signals” approach operationalizes the mechanistic story at the bedside.

Risk is not uniform across populations. Young-adult cohorts show nontrivial rates of PSE after both ischemic stroke and ICH, with distinct clinical correlates that should inform surveillance and counseling [1]. In large prospective datasets from Asia, post-stroke seizures are associated with increased mortality and worse functional recovery, emphasizing the global relevance of PSE and the need for context-specific tools and resources [34]. ICH cohorts from different regions have yielded prediction tools tailored to hematoma patterns, and these may complement or refine the CAVE family in specific clinical contexts [35]. SAH, with its distinct mechanisms (cortical irritation from subarachnoid blood, vasospasm, delayed cerebral ischemia), calls for targeted tools like RISE and for attention to CSF inflammatory profiles [19,24].

Genetics and polygenics further stratify risk. Systematic reviews identify polymorphisms in inflammatory and synaptic genes associated with seizures after acute brain injury, while biobank-scale analyses link higher epilepsy polygenic risk to PSE susceptibility in stroke survivors [29,30]. These observations argue for a precision-medicine layer built atop clinical scores and dynamic biomarkers, particularly as the cost and logistics of genotyping continue to fall.

Linking mechanism, measurement, and management: a synthesis

The through-line from injury to epilepsy begins with excitotoxic and metabolic crises that trigger BBB leak, glymphatic dysfunction, and innate immune activation; it continues through epigenetic and transcriptional reprogramming that reshapes channel expression and synaptic composition; and it culminates in maladaptive sprouting, interneuron loss, and network synchronization. Along this pipeline, electrographic instability and perfusion/metabolic signatures serve as translational proxies for the evolving substrate. Imaging captures lesion topology and microstructural trajectories; EEG reveals the real-time dynamics of cortical networks; blood and CSF markers report on glial activation, BBB integrity, and inflammation. The most compelling preventive strategies are those that map cleanly onto this sequence and that can be monitored with congruent surrogate endpoints: IL-1-pathway antagonism tracked by cytokine panels and interictal burden, ROCK2-mitophagy restoration followed by SV2A PET and qEEG connectivity, and systems-level sleep and vascular interventions indexed by improvements in overnight EEG stability and inflammatory markers [10-15,20-22].

Comparison of epileptogenesis between post-ischemic and post-hemorrhagic seizures

Although ischemic and hemorrhagic strokes share many downstream epileptogenic mechanisms, including neuronal loss, gliosis, BBB dysfunction, and maladaptive synaptic remodeling, the relative contributions and temporal profiles of these processes differ between etiologies. In ischemic stroke, early excitotoxicity and metabolic failure within the infarct core and penumbra dominate the acute phase, with subsequent epileptogenesis shaped by peri-infarct cortical reorganization, interneuron loss, and evolving small-vessel and sleep-glymphatic comorbidities. By contrast, in intracerebral hemorrhage, extravasated blood and its breakdown products provide an immediate source of cortical irritation and oxidative stress, while the resolving hematoma leaves a persistent hemosiderin-rich, gliotic scar that serves as a long-term epileptogenic nidus [17-22].

Clinically, seizures occur more frequently and earlier after hemorrhagic than ischemic stroke, and lobar cortical hematomas carry the highest risk of both acute symptomatic and late seizures. In ischemic cohorts, risk is concentrated among patients with large anterior-circulation infarcts, cortical involvement, hemorrhagic transformation, and early seizures, and is well captured by SeLECT family models. In hemorrhagic cohorts, CAVE and modified CAVE scores quantify how cortical location, hematoma volume, younger age, and early seizures interact to determine late seizure risk. At the molecular level, both etiologies engage overlapping inflammatory, oxidative, and epigenetic programs, but the upstream triggers differ: ischemia-reperfusion and metabolic crisis in infarction versus blood-product toxicity and clot resolution in ICH. Experimental and clinical data suggest that these differences may modulate the tempo and spatial distribution of BBB disruption, glial activation, and synaptic reorganization, with potential implications for the timing and choice of anti-epileptogenic interventions. Comparative studies explicitly contrasting biomarker trajectories and network evolution after ischemic versus hemorrhagic stroke remain limited and represent a key area for future work [20-22].

To further underscore the etiologic heterogeneity of post-stroke epileptogenesis, we summarize key differences between ischemic and hemorrhagic stroke in Table 1. This comparison highlights distinct upstream triggers and structural consequences, despite convergence onto partially overlapping inflammatory and network-remodeling pathways.

**Table 1:** Key differences in epileptogenesis after ischemic versus hemorrhagic stroke Source: [17-22].

Feature	Post-ischemic stroke epileptogenesis	Post-hemorrhagic stroke epileptogenesis	Clinical implications
Primary initiating insult	Focal arterial occlusion → ischemia, energy failure, excitotoxicity in infarct core/penumbra	Parenchymal blood extravasation (ICH) or subarachnoid blood → mass effect, mechanical cortical irritation, blood-product toxicity	Different upstream triggers but convergent downstream pathways (gliosis, BBB leak, inflammation)
Dominant acute drivers	Glutamate-mediated excitotoxicity, ionic disequilibria, mitochondrial dysfunction, early innate immune activation	Hemoglobin/iron toxicity, thrombin and other clot-related mediators, perihematomal edema, abrupt BBB disruption	May influence optimal timing of neuroprotective and anti-inflammatory interventions
Structural evolution	Peri-infarct cortical thinning and gliosis; maladaptive sprouting in peri-infarct cortex and hippocampus	Gliotic, hemosiderin-laden cavity at cortical-subcortical interface; chronic hemosiderosis around hematoma bed	Chronic irritative zone more spatially circumscribed in lobar ICH; more diffuse network remodeling in large infarcts
BBB and glymphatic changes	Gradual BBB breakdown and impaired glymphatic clearance in peri-infarct regions; interaction with small-vessel disease and sleep fragmentation	Marked, focal BBB disruption adjacent to hematoma; edema and mass effect perturb local perivascular/glymphatic flow	Shared therapeutic targets (sleep, vascular risk factor control), but with differing topographies of BBB injury
Typical seizure timing	Early seizures approximately 5%-6% of patients; late PSE in approximately 3%-7%, concentrated in large cortical/anterior-circulation infarcts	Early seizures more frequent (approximately 5%-14%); late seizures in approximately 8%-10% of ICH survivors, especially lobar/cortical ICH	Higher baseline seizure risk after lobar ICH; closer early EEG surveillance often justified
Key clinical risk factors	Large lesion volume, high NIHSS, cortical/anterior-circulation involvement, hemorrhagic transformation, early seizures, younger age, some genetic variants	Lobar cortical location, larger hematoma volume, intraventricular extension, early seizures, younger age	Inform SeLECT-family (ischemia) and CAVE-family (ICH) risk scores and selection of high-risk patients for trials
Representative prediction tools	SeLECT and SeLECT 2.0 (late seizure risk after ischemic stroke)	CAVE and modified CAVE scores (late seizure risk after ICH)	Provide pragmatic, etiology-specific frameworks for monitoring and for biomarker-enriched trial design

Future directions

What would a next-generation preventive trial look like? First, eligibility would be biomarker-enriched: large cortical infarcts or lobar ICH (per SeLECT 2.0 or modified CAVE thresholds) plus early electrographic abnormalities and elevated glial/inflammatory markers during the first week. Second, therapy would be mechanism-matched and phase-aware, for example, an IL-1 pathway antagonist initiated within days of stroke or a mitophagy-restoring agent started in the subacute latent period when synaptic consolidation is underway. Third, the primary endpoints would be composite surrogates (reduction in interictal burden, normalization of GFAP/IL-1β trajectories, improvement in qEEG connectivity indices) measured serially over 12-16 weeks, with secondary clinical endpoints of time to first late seizure and 12-24-month epilepsy incidence. Finally, trial infrastructure would harmonize EEG acquisition, sampling schedules, and analytic pipelines to maximize signal and generalizability across centers.

Parallel agendas should include: (1) deeper phenotyping of sleep and CSVD in post-stroke cohorts to refine the BBB-glymphatic narrative; (2) validation of miRNA and metabolomic signatures that can be deployed in routine care; (3) prospective, cross-platform validation of ML predictors that combine clinical scores, imaging, EEG, and biomarkers; and (4) careful exploration of non-pharmacologic neuromodulation and conditioning paradigms with mechanistic endpoints [26-28,33]. Above all, preclinical models must better mirror human stroke biology age, comorbidity, polypharmacy, and lesion heterogeneity so that mechanistic leads and dosing regimens translate more reliably.

## Conclusions

Epileptogenesis after stroke should be viewed as a long, dynamic cascade rather than a single event. Structural damage and molecular disturbances unfold together: cell loss, gliosis, BBB leakage, and progressive network reshaping interact with inflammatory signaling, excitotoxic injury, oxidative stress, and epigenetic shifts. Together, these changes gradually create a brain that is more susceptible to seizures. Recent work has produced encouraging leads: candidate biomarkers from imaging, electrophysiology, and biofluids, as well as clinical tools that estimate risk based on lesion characteristics and patient factors. Still, we have not yet reached the point where early prevention is practical. Predictive models remain insufficiently validated, and trials of anti-epileptogenic therapies have struggled without mechanism-specific markers to track biological response. To advance the field, future studies must deliberately align trial design with the stepwise biology of epileptogenesis. This includes using biomarker-enriched cohorts and repeated, multimodal measurements that correspond to particular mechanistic stages. Integrating polygenic risk, machine-learning prediction, and post-stroke care pathways that blunt prolonged inflammation and metabolic dysfunction will further refine prevention strategies. If these efforts succeed, clinical practice can shift from treating seizures after they emerge to preventing them altogether, ultimately improving long-term outcomes and quality of life for people recovering from stroke.

## References

[1] Verburgt E, Fellah L, Ekker MS (2025). Risk of poststroke epilepsy among young adults with ischemic stroke or intracerebral hemorrhage. JAMA Neurol.

[2] Misra S, Kasner SE, Dawson J (2023). Outcomes in patients with poststroke seizures: a systematic review and meta-analysis. JAMA Neurol.

[3] Greenberg SM, Ziai WC, Cordonnier C (2022). 2022 guideline for the management of patients with spontaneous intracerebral hemorrhage: a guideline from the American Heart Association/American Stroke Association. Stroke.

[4] Pozo Putalivo JA, Fariña S, Sol P, Grecco M, Sampaio M, Povedano GP (2025). Identifying clinical and imaging predictors of post stroke epilepsy. Epilepsy Behav.

[5] Hussein MA, Kiwan NA, Aly YR, Badawy AA, Hussein AM (2025). Role of hippocampus in epileptogenesis: new insights in the cross-talks between the underlying mechanisms. Acta Neurol Belg.

[6] Li Y, Su Z, Zhao K, Liu X, Chen S, Yang X, Zhou L (2025). Epigenetic mechanisms in the pathophysiology and progression of epilepsy: a comprehensive review of experimental and clinical studies. Curr Neuropharmacol.

[7] Bitar R, Khan UM, Rosenthal ES (2024). Utility and rationale for continuous EEG monitoring: a primer for the general intensivist. Crit Care.

[8] Hirsch LJ, Fong MW, Leitinger M (2021). American Clinical Neurophysiology Society's Standardized Critical Care EEG Terminology: 2021 Version. J Clin Neurophysiol.

[9] Gururangan K, Kozak R, Dorriz PJ (2025). Time is brain: detection of nonconvulsive seizures and status epilepticus during acute stroke evaluation using point-of-care electroencephalography. J Stroke Cerebrovasc Dis.

[10] Dev P, Cyriac M, Chakravarty K, Pathak A (2022). Blood and CSF biomarkers for post-stroke epilepsy: a systematic review. Acta Epileptologica 4.

[11] Ryu HU, Kim HJ, Shin BS, Kang HG (2024). Clinical approaches for poststroke seizure: a review. Front Neurol.

[12] Hein ZM, Al-Zaghal ZA, Muhammad Ghazali M (2025). Mechanistic insights into the sleep-glymphopathy-cerebral small vessel disease loop: implications for epilepsy pathophysiology and therapy. Front Neurosci.

[13] Chen T, Wen S, Gong L, Hou X, Dong L, Bai Y, Xu Z (2025). Lactylation as a metabolic-epigenetic nexus in epilepsy: mechanisms and therapeutic implications. Neurobiol Dis.

[14] Xiao L, Wang J, Chen B (2025). Targeting ROCK2 to restore epileptic synaptic networks via mitophagy activation: Insights from Translational Imaging of SV2A In Vivo. Adv Sci (Weinh).

[15] Tada Y, Fujihara T, Yamaguchi I (2024). Nonconvulsive status epilepticus in patients with acute subarachnoid hemorrhage is associated with negative arterial spin labeling on peri-ictal magnetic resonance images. Heliyon.

[16] Lin J, Li X, Ye J, Li G, Huang S, Zhu S (2023). External validation and comparison of clinical scores for predicting late seizures after intracerebral hemorrhage in Chinese patients. Epilepsy Behav.

[17] Huang YC, Wong YS, Wu CS, Tsai CF, Ong CT (2023). Modified CAVE score for predicting late seizures after intracerebral hemorrhage. BMC Neurol.

[18] Meletti S, Cuccurullo C, Orlandi N (2024). Prediction of epilepsy after stroke: proposal of a modified SeLECT 2.0 score based on posttreatment stroke outcome. Epilepsia.

[19] Campos-Fernandez D, Rodrigo-Gisbert M, Abraira L (2024). Predictive model for estimating the risk of epilepsy after aneurysmal subarachnoid hemorrhage. The RISE score. Neurology.

[20] Schubert KM, Dasari V, Oliveira AL (2025). The role of electroencephalography in predicting post-stroke seizures and an updated prognostic model (SeLECT-EEG). Ann Neurol.

[21] Tatillo C, Legros B, Depondt C (2024). Prognostic value of early electrographic biomarkers of epileptogenesis in high-risk ischaemic stroke patients. Eur J Neurol.

[22] Furlanis G, Ricci E, Ajčević M (2025). Early point-of-care EEG in acute stroke: prevalence and predictive factors of early post-stroke status epilepticus (e-PSSE). Seizure.

[23] Zhang L, Zheng W, Chen F, Bai X, Xue L, Liang M, Geng Z (2021). Associated factors and prognostic implications of non-convulsive status epilepticus in ischemic stroke patients with impaired consciousness. Front Neurol.

[24] Tian F, Liang J, Liu G, Zhang X, Cai Z, Huo H, Chai E (2021). Postinfectious inflammation in cerebrospinal fluid is associated with nonconvulsive seizures in subarachnoid hemorrhage patients. Epilepsy Res.

[25] Wang N, Wang D, Zhou H, Xu C, Hu X, Qian Z, Xu X (2021). Serum neuropeptide Y level is associated with post-ischemic stroke epilepsy. J Stroke Cerebrovasc Dis.

[26] Lekoubou A, Petucci J, Ajala TF, Katoch A, Sen S, Honavar V (2024). Large datasets from electronic health records predict seizures after ischemic strokes: a machine learning approach. medRxiv.

[27] Lekoubou A, Petucci J, Femi Ajala T (2024). Can machine learning predict late seizures after intracerebral hemorrhages? Evidence from real-world data. Epilepsy Behav.

[28] Schubert KM, Schmick A, Stattmann M, Galovic M (2025). Prognostic models for seizures and epilepsy after stroke, tumors and traumatic brain injury. Clin Neurophysiol Pract.

[29] Misra S, Quinn TJ, Falcone GJ (2023). Impact of genetic polymorphisms on the risk of epilepsy amongst patients with acute brain injury: a systematic review. Eur J Neurol.

[30] Clocchiatti-Tuozzo S, Rivier CA, Misra S (2024). Polygenic risk of epilepsy and poststroke epilepsy. Stroke.

[31] Frontera JA, Rayi A, Tesoro E (2025). Guidelines for seizure prophylaxis in patients hospitalized with nontraumatic intracerebral hemorrhage: a clinical practice guideline for health care professionals from the Neurocritical Care Society. Neurocrit Care.

[32] Peter-Derex L, Philippeau F, Garnier P (2022). Safety and efficacy of prophylactic levetiracetam for prevention of epileptic seizures in the acute phase of intracerebral haemorrhage (PEACH): a randomised, double-blind, placebo-controlled, phase 3 trial. Lancet Neurol.

[33] Alhashimi A, Kamarova M, Baig SS (2024). Remote ischaemic conditioning for neurological disorders-a systematic review and narrative synthesis. Syst Rev.

[34] Freiman S, Hauser WA, Rider F (2023). Post-stroke seizures, epilepsy, and mortality in a prospective hospital-based study. Front Neurol.

[35] Wang Y, Li Z, Zhang X (2021). Development and validation of a clinical score to predict late seizures after intracerebral hemorrhage in Chinese. Epilepsy Res.

